# ﻿Two new species of *Cytospora* (Diaporthales, Cytosporaceae) causing canker disease of *Malusdomestica* and *M.sieversii* in Xinjiang, China

**DOI:** 10.3897/mycokeys.109.131456

**Published:** 2024-10-16

**Authors:** Guifang Cai, Ying Zhao, Yawei Zhai, Meilin Yan, Rong Ma, Daoyuan Zhang

**Affiliations:** 1 College of Forestry and Landscape Architecture, Xinjiang Agricultural University, Urumqi, 830052, China Xinjiang Agricultural University Urumqi China; 2 Forestry and Grassland Technology Extension Center of Changji Prefecture, Changji 831100, China Forestry and Grassland Technology Extension Center of Changji Prefecture Changji China; 3 Forestry and Grassland Resources Monitoring Center of Xinjiang Production and Construction Corps, Urumqi, 830002, China Forestry and Grassland Resources Monitoring Center of Xinjiang Production and Construction Corps Urumqi China; 4 China Energy Engineering Group Xin Jiang Electric Power Design Institute CO., LTD., Urumqi, 830050, China China Energy Engineering Group Xin Jiang Electric Power Design Institute CO., LTD. Urumqi China; 5 Forestry and Grassland Bureau of Hinggan League, Hinggan, 137599, China Forestry and Grassland Bureau of Hinggan League Hinggan China; 6 CAS Key Laboratory of Biogeography and Bioresource in Arid Land, Xinjiang Institute of Ecology and Geography, Urumqi, China CAS Key Laboratory of Biogeography and Bioresource in Arid Land, Xinjiang Institute of Ecology and Geography Urumqi China

**Keywords:** Apple, Ascomycota, phylogeny, plant disease, taxonomy, wild apple

## Abstract

Apple tree canker is a serious disease caused by species of *Cytospora*. Xinjiang Uygur Autonomous Region is one of the most important apple-producing areas in China. However, losses due to apple *Cytospora* canker have seriously damaged the apple industry and affected the economic development of the apple growers in this region. In this study, we used morphological characteristics combined with multilocus phylogenetic analyses of the ITS, *act*, *rpb2*, *tef1* and *tub2* loci to identify isolates from apple (*Malusdomestica*) and wild apple (*M.sieversii*). As a result, *C.hippophaopsis***sp. nov.** from *M.sieversii and C.shawanensis***sp. nov.** from *M.domestica* were discovered and proposed herein. Pathogenicity tests were further conducted on 13 varieties of apple and wild apple, which confirmed *C.hippophaopsis* and *C.shawanensis* as canker pathogens. Meanwhile, *C.hippophaopsis* is generally more aggressive than *C.shawanensis* on the tested varieties of apple and wild apple.

## ﻿Introduction

Wild apple (*Malussieversii*) is a tertiary relict plant distributed only in the Tianshan mountain of central Asia. It is located in Emin and Yumin Counties of Tacheng area, Xinyuan, Gongliu and Huocheng Counties of Ili area in Xinjiang Uygur Autonomous (XJUA) Region, China. It is rich in intraspecific variation and has strong resistance, high yield, dwarf and other characters, and is often used in rootstock grafting, cross breeding, and is also important for introduction, domestication and germplasm resources (Richards et al. 2009; Volk et al. 2013; Wang et al. 2018). It plays an irreplaceable role in the study of germplasm conservation and genetic development (Yan et al. 2008; Richards et al. 2009; Volk et al. 2013). This species has been listed in the index of China’s endangered secondary protected plants (Fu 1991). Apple (*M.domestica*) is one of the four major fruits in the world and one of the 11 major agricultural products identified by the Ministry of Agriculture of China. XJUA Region is one of eight high-yield apple production bases in China, accounting for more than 3.5% of production. The cultivation has a long history, due to adequate sunshine, high effective accumulated temperature, large temperature difference between day and night (Li et al. 2015; Snyder and Ni 2017). Apple tree canker has been reported as a common disease in Tekes County of Ili area in XJUA Region and the death rate of apple trees reached 30% to 40%, which caused great loss to the apple industry (Yang 2009).

More than 20 species of *Cytospora* have been reported on *Malus* spp. worldwide (Zhuang 2005; Ma et al. 2018; Azizi et al. 2020; Wang et al. 2020; Bozorov et al. 2024), of these *C.leucostoma*, *C.mali*, *C.parasitica* and *C.schulzeri* have been reported to infect *M.domestica* in XJUA Region (Zhuang 2005; Ma et al. 2018; Liu et al. 2020). Meanwhile, three species of *Cytospora* were recorded on *M.sieversii*, viz. *C.leucostoma* and *C.mali* distributed in Gongliu, Aksu, Urumqi, Turpan and Shanshan (Zhuang 2005), and *C.parasitica* distributed in Tacheng and Ili areas (Ma et al. 2018).

The genus *Cytospora* (Cytosporaceae, Diaporthales) comprises many important phytopathogens that cause dieback and canker disease on twigs, branches and stems of various woody species and can cause large areas of dieback on a wide range of plants, resulting into severe commercial and ecological damage and significant losses worldwide (Adams et al. 2005; Senanayake et al. 2017; Norphanphoun et al. 2018; Fan et al. 2020; Jiang et al .2020; Li et al. 2024).

*Cytospora* was initially introduced with *C.betulina*, *C.epimyces*, *C.resinae* and *C.ribis* in 1818, and *C.chrysosperma* was added to this genus and subsequently selected as the type species (Ehrenberg 1818; Fries 1823; Donk 1964). Species of this genus are characterized by having allantoid hyaline spores in both sexual and asexual states (Zhu et al. 2019; Fan et al. 2020; Pan et al. 2020, 2021; Lin et al. 2023a, 2023b). Species identification in *Cytospora* was previously based on their hosts and morphological characters (Donk 1964; Ehrenberg 1818; Fries 1823). Molecular phylogeny based on sequence data has been applied to separate species of this genus recently (Adams et al. 2005; Fan et al. 2015; Wang et al. 2015; Norphanphoun et al. 2017; Lawrence et al. 2018).

Canker diseases caused by *Cytospora* species are leading to serious economic losses in apple plantations of XJUA Region in China. The aims of this study were to clarify the taxonomy statues of the newly collected species from the host genus *Malus* in this region, and to test their pathogenicity.

## ﻿Materials and methods

### ﻿Sample collection, isolation and morphology

**Figure 1.**
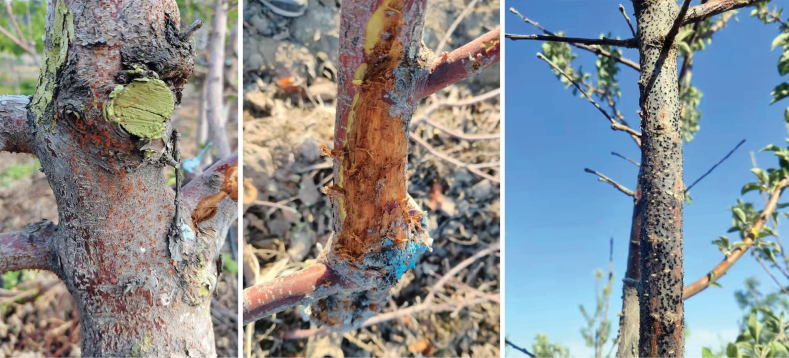
Symptoms of apple tree cankers in Xinjiang, China.

*Malus* canker disease investigations were conducted during 2015 and 2022 in XJUA Region. Trees with dead stems, branches and twigs were checked, and fruiting bodies were discovered on the tree barks (Fig. 1). Samples were packed in paper bags and turned to laboratory for further research.

Ascomata and conidiomata formed on barks of *Malusdomestica* and *M.sieversii* were sectioned using a sterile blade, and mucoid spore masses were removed to the surface of the potato dextrose agar (PDA; potato, 200 g; glucose, 20 g; agar, 20 g; distilled water, to complete 1000 mL) media using a sterile needle. Then plates were incubated at 25 °C in darkness until spores germinated. Pieces of mycelium were cut and removed to a new PDA plate under a stereomicroscope to obtain the pure cultures.

The new species of *Cytospora* were observed mainly based on the fruiting bodies naturally formed on the tree barks. The ascomata and conidiomata were sectioned using a sterile blade and photographed under the Leica stereomicroscope (M205) (Leica Microsystems, Wetzlar, Germany). The asci, ascospores, conidiophores, conidiogenous cells and conidia were measured and photographed by a Nikon Eclipse 80i microscope (Nikon Corporation, Tokyo, Japan). The colony characters were observed and recorded on PDA plates at 25 °C under darkness.

### ﻿DNA extraction, PCR amplification and phylogenetic analyses

The total DNA of strains of *Cytospora* collected in the present study was extracted from cultures growing on the PDA plates overlaid with cellophane based on the CTAB method (Doyle 1990). Five primer pairs ITS1/ITS4, ACT512F/ACT783R, fRPB2-5f/fRPB2-7cR, 983F/2218R and Bt2a/Bt2b were used to amplify the internal transcribed spacer region rDNA (ITS), the partial actin (*act*) region, RNA polymerase II second largest subunit (*rpb2*), translation elongation factor 1-alpha (*tef1*) and the partial beta-tubulin (*tub2*) gene loci, respectively (White et al. 1990; Glass and Donaldson 1995; Carbone and Kohn 1999; Liu et al. 1999; Rehner 2001). The polymerase chain reaction (PCR) conditions were as performed as follows: an initial denaturation step of 5 min at 94 °C, followed by 35 cycles of 30 s at 94 °C, 50 s at 52 °C (ITS), 54 °C (*tef1* and *tub2*), 55 °C (*rpb2*) or 58 °C (*act*), and 1 min at 72 °C, and a final elongation step of 7 min at 72 °C. All amplified PCR products were estimated visually with 1.4% agarose gels stained with ethidium bromide and then PCR positive products were sent to Sangon Biotech (Shanghai) Co., Ltd., (Beijing, China) for sequencing.

Sequences obtained in the present study were preliminary identified by the BLAST search to confirm their classification. The referenced sequences of *Cytospora* were collected from Wang et al. (2024) and downloaded. *Diaporthevaccinii* (CBS 160.32) was selected as the outgroup taxon. The five individual loci ITS, *act*, *rpb2*, *tef1* and *tub2* were aligned using MAFFT v. 6.0 and edited manually using MEGA v. 6.0 (Katoh and Standley 2013; Tamura et al. 2013). Then five loci were combined and analyzed based on maximum likelihood (ML) and Bayesian inference in the CIPRES Science Gateway platform (Miller et al. 2010). The GTR substitution model were employed, and 1000 non-parametric bootstrap replicates were set for ML phylogenic analysis. Four simultaneous Markov Chain runs for 1000000 generations were set during Bayesian analysis. The resulting trees were visualized in FigTree v. 1.4.0 and edited by Adobe Illustrator 2020.

### ﻿Pathogenecity tests

To determine the pathogenicity of *C.hippophaopsis* and *C.shawanensis* newly proposed in the present study to 13 apple varieties (*Malusdomestica*) and wild apple (*M.sieversii*). Healthy branches were collected from three-year-old healthy trees of Red Meat Apple (RMA), Jonagold (JNG), Golden Delicious (GD), Manpanzi (MPZ), Sitagan (STG), Oil fruit (OF), Red Star (RS), Fuji (FU), Erzizi (EZ), Qiulimeng (QM), Apolte (AP), Hanfu (HF), New Century (NC) and wild apple *M.sieversii* (MS) in Ili Kazakh Autonomous Prefecture in XUAR in July 2019. The branches were washed with water and disinfected with a 75% (vol/vol) ethanol solution, then branches were cut into 30-cm long segments. Both ends of the branch segments were sealed with paraffin wax to reduce desiccation. A soldering iron was used to create a wound 1 mm in diameter a third of the way along each branch segment and leaf. A 3-mm-diameter mycelial plug which cultured for three days of *C.hippophaopsis* and *C.shawanensis* species was placed onto the wound of branches. There were three replicates per treatment for branch tests. The materials of branch that inoculated with sterile PDA plugs were used as controls. All tested materials were incubated in humid chambers at room temperature (26 ± 2 °C) in12 h light and 12 h dark. The mycelial plug was removed after 24 hours. The virulence of *C.hippophaopsis* and *C.shawanensis* was assessed by measuring the lesion length (L) and width (W) of the wounded twig segments every 2 days for 30 days to determine the lesion area (mm^2^). The lesion area (S) of each replicate was calculated using the following formula: S = π × L/2 × W/2. Re-isolations were conducted from the tested branches, and the reisolates were identified based on colony characteristics and molecular data.

The data were analyzed and figured using R (Version 4.3.3, R core team, Viena). Two-way analysis of variance (ANOVA) was performed based on the lesion area data by taking *Cytospora* species and varieties of apple tree and wild apple as fixed factors. Post hoc test of Tukey’s least significant difference was carried out by p < 0.05. The dominant packages used were “emmeans”, “tidyverse”, and “ggplot2”.

## ﻿Results

### ﻿Phylogeny

The combined sequence dataset of ITS, *act*, *rpb2*, *tef1* and *tub2* consisted of 202 strains with *Diaporthevaccinii* (CBS 160.32) as the outgroup taxon. In the final alignment, there are 556 characters in ITS, 324 characters in *act*, 740 characters in *rpb2*, 735 characters in *tef1* and 839 characters *tub2*. The final ML optimization likelihood value of the best RAxML tree was -60551.35, and the matrix had 2092 distinct alignment patterns, with 40.58% undetermined characters or gaps. Estimated base frequencies were as follows: A = 0.244681, C = 0.288135, G = 0.237104, T = 0.230081; substitution rates AC = 1.269712, AG = 2.848941, AT = 1.301835, CG = 0.952261, CT = 4.964970, GT = 1.0; gamma distribution shape parameter α = 0.377319. The topologies resulting from ML and BI analyses of the concatenated dataset were congruent (Fig. 1). Two isolates named XJAU 1378 and XJAU 1379 from *Malussieversii* clustered into a distinct clade in the phylogram. Meanwhile, XJAU 866 and XJAU 867 from *M.domestica* formed another clade different from any known species (Fig. 1).

### ﻿Taxonomy

#### ﻿ Cytospora hippophaopsis

Taxon classificationFungiDiaporthalesCytosporaceae

R. Ma & G.F. Cai sp. nov.

74FC5144-8E6A-53E0-879C-92886EC9F2CA

 825187

Fig. 3

##### Etymology.

Referring to its morphological similarity to *C.hippophaës*.

##### Descriptions.

Ascostromata erumpent through the surface of bark. Disc black and circular with 6–16 ostioles per disc, ostiole black and circular, 54.5–103 µm (av. = 78.8 µm, n = 30) diam. Perithecium globular, 180.5–395 µm (av. = 285.1 µm, n = 30) diam. Asci clavate to elongate obovoid, 59.5–87 × 8–12.5 µm (av. = 73.6 × 9.9 µm, n = 30), eight-spored. Ascospores biseriate, elongate-allantoid, hyaline thin-walled and smooth-walled, 13–22.5 × 3.5–5.5 µm (av. = 18.4 × 4.4 µm, n = 50).

##### Cultural characteristics.

Colonies on PDA initially white, covering the petri dish in 7 d, becoming brown after 14 d. Ascomata formed after 16 d, white at the early stage, becoming black later.

##### Material examined.

China • Xinjiang Uygur Autonomous, Ili Autonomous Prefecture, Gongliu County, KurdinNing, Xiaomohe, 43°11'44.15"N, 82°43'41.66"E, 1288 m asl, on dead and dying branches of *Malussieversii*, Rong Ma, 4 Aug 2016, holotype XJAU-1378, living cultures XJAU 1378 = CGMCC 3.18997 (ITS: PP965505, *act*: PP957863, *rpb2*: PP957870, *tef1*: PP957877, *tub2*: PP957884); *ibid*. XJAU 1379 (ITS: PP965506, *act*: PP957864, *rpb2*: PP957871, *tef1*: PP957878, *tub2*: PP957885).

##### Notes.

**Figure 2.**
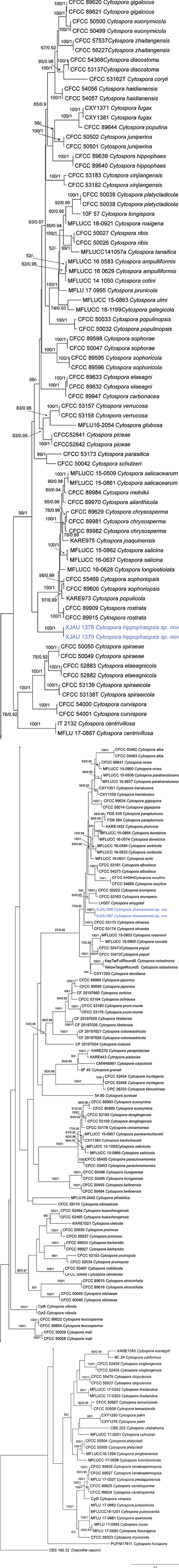
Phylogenetic tree of *Cytospora* of ML analysis on basis of combined ITS, *act*, *rpb2*, *tef1* and *tub2* loci. Numbers above the branches indicate ML bootstraps (left, ML BS ≥ 50%). The tree is rooted with *Diaporthevaccinii* (CBS 160.32). New species from the present study are marked in blue.

Two isolates (XJAU 1378 and XJAU 1379) from the present study formed a distinct clade in the genus *Cytospora* (Fig. 2), which represents a new species named *C.hippophaopsis*. *Cytosporahippophaopsis* is morphologically similar to *C.hippophaës* from *Hippophaërhamnoides* but differs in larger asci (59.5–87 × 8–12.5 µm vs. 38.6–44 × 5.9–7.8 µm) and ascospores (13–22.5 × 3.5–5.5 µm vs. 11.8–15 × 3–4.1 µm) (Fan et al. 2015).

**Figure 3.**
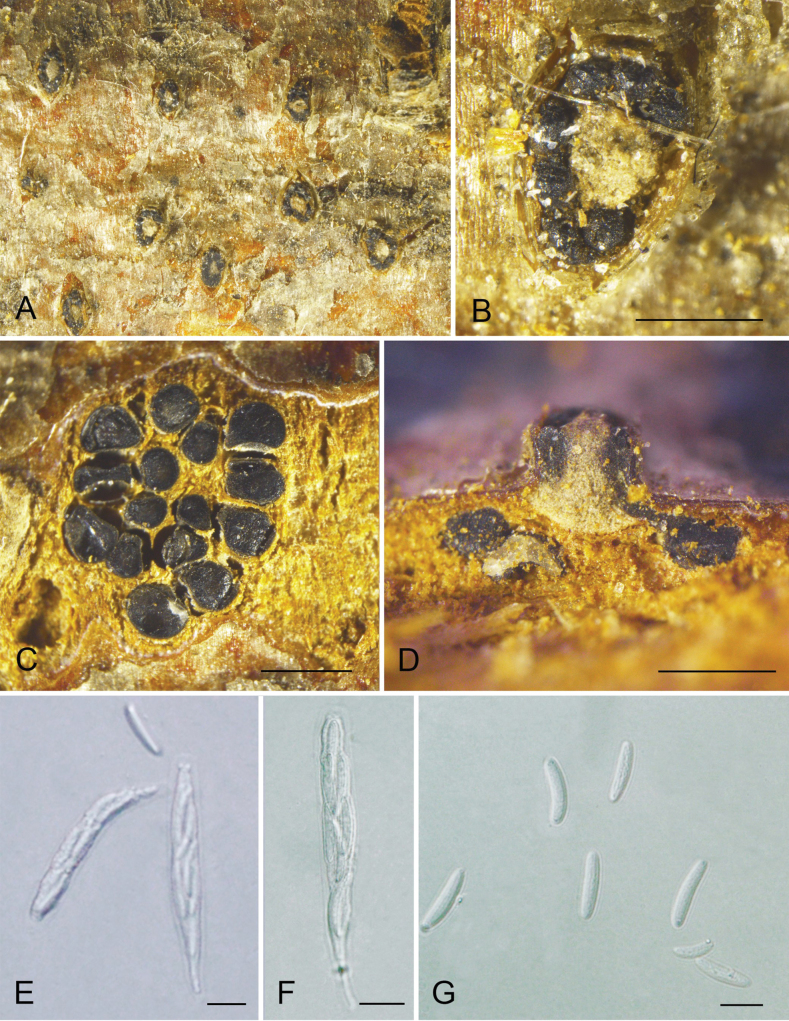
Morphology of *Cytosporahippophaopsis* (XJAU 1378) **A, B** habitat of ascostromata on the host bark **C** transverse section of the ascostromata **D** longitudinal section through the ascostromata **E–G** asci and ascospores. Scale bars: 300 µm (**B–D**); 10 µm (**E–G**).

#### ﻿ Cytospora shawanensis

Taxon classificationFungiDiaporthalesCytosporaceae

R. Ma & G.F. Cai sp. nov.

0CBFF44B-4D49-5BF8-A069-4D3D9CAB1288

 825189

Fig. 4

##### Etymology.

Named after the collection site, Shawan.

##### Descriptions.

Stromata pycnidial, ostiolate, immersed in the host bark, scattered, producing black area on bark, circular to ovoid, with multiple locules. Conceptacle absent. Ectostromatic disc inconspicuous, producing one ostiole per disc when mature. Ostiole in the centre of the disc, black, conspicuous, 60–190 μm diam. Locules numerous, arranged circularly or elliptically with independent walls, 460–1420 μm diam. Conidiophores hyaline, unbranched or branched at the bases. Conidiogenous cells enteroblastic, phialidic, smooth-walled, tapering towards apex. Conidia hyaline, unicellular, smooth-walled, 3.5–5.5 × 1–1.5 µm (av. = 4.3 × 1.2 µm, n = 50).

**Figure 4.**
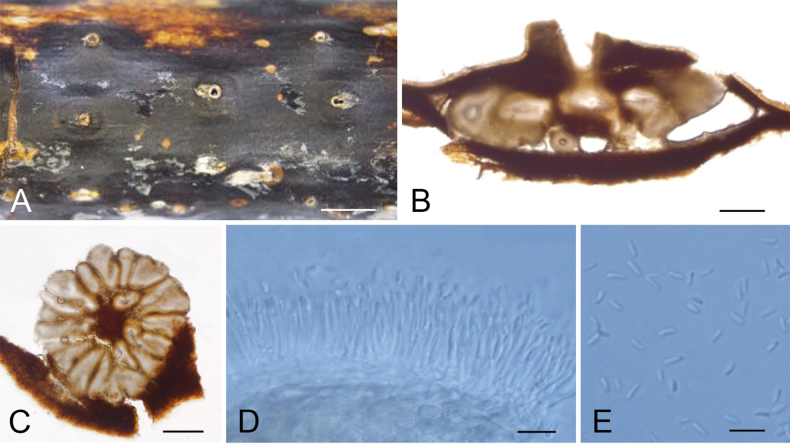
Morphology of *Cytosporashawanensis* (XJAU 866) **A** habitat of conidiomata on the host branch **B** longitudinal section of the conidioma **C** transverse section through the conidioma **D** conidiogenous cells with attached conidia **E** conidia. Scale bars: 1000 µm (**A**); 200 µm (**B, C**); 10 µm (**D, E**).

##### Cultural characteristics.

Colonies on PDA initially white, covering the petri dish in 3 d, becoming grayish green after 14 d. Conidiomata formed after 5 d, randomly distributed in the colony.

##### Material examined.

China • Xinjiang Uygur Autonomous, Tacheng City, Shawan County, Daquan Village, 44°20'1.24"N, 85°37'34.40"E, 528 m asl, on dead and dying branches of *Malusdomestica*, Rong Ma, 28 Jul 2015, holotype XJAU-866, living cultures XJAU 866 = CGMCC 3.18996 (ITS: PP965507, *act*: PP957865, *rpb2*: PP957872, *tef1*: PP957879, *tub2*: PP957886); *ibid*. XJAU 867 (ITS: PP965508, *act*: PP957866, *rpb2*: PP957873, *tef1*: PP957880, *tub2*: PP957887).

##### Notes.

*Cytosporashawanensis* is phylogenetically close to *C.olivacea* in the phylogram (Fig. 2). These two species share similar conidial morphology but differs in the sequence data (2/502 bp in ITS, 20/249 bp in *act*, 41/726 bp in *rpb2* and 39/375 bp in *tub2*). In addition, host association is helpful for species identification (*C.shawanensis* on *Malusdomestica vs. C.olivacea* on *Sorbustianschanica*) (Pan et al. 2020).

### ﻿Pathogenecity tests

**Figure 5.**
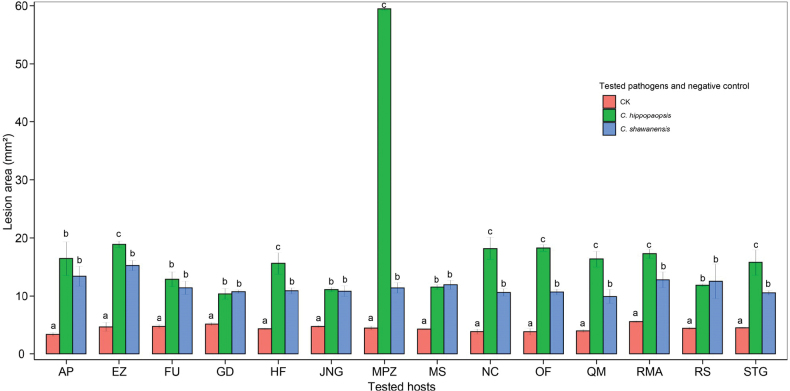
Pathogenicity of different *Cytosporahippophaopsis* and *C.shawanensis* on 13 varieties of apple (*Malusdomestica*) and wild apple (*M.sieversii*). Values are presented as means ± SE of three replicates. Different lowercase letters in bracket indicate significant differences among apple varieties and wild apple (*p < 0.05*). Apple varieties: AP, Apolte; EZ, Erzizi; FU, Fuji; GD, Golden Delicious; HF, Hanfu; JNG, Jonagold; MPZ, Manpanzi; NC, New Century; OF, Oil Fruit; QM, Qiulimeng; STG, Sitagan; RMA, Red Meat Apple; RS, Red Star. Wild apple: MS, *M.sieversii*.

After 30 days of incubation on apple and wild apple branches, re-isolates were obtained from the tested tissues and identified. The *Cytospora* species from the tested tissues were the same as those used for incubation, both phylogenetically and morphologically, whereas no *Cytospora* species were obtained from negative controls. As shown in the Fig. 5, *Cytosporahippophaopsis* and *C.shawanensis* create obviously larger lesion areas on apple (*Malusdomestica*) and wild apple (*M.sieversii*) than that by negative control. Hence, *C.hippophaopsis* and *C.shawanensis* are both considered as *Malus* canker pathogens. Generally, *C.hippophaopsis* is more aggressive than *C.shawanensis* for the obviously larger lesion areas on Erzizi, Hanfu, Manpanz, New Century, Oil Fruit, Sitagan and Red Star. Meanwhile, these two pathogens share similar virulence to Apolte, Fuji, Golden Delicious, Jonagold, Red Star of apple and wild apple.

## ﻿Discussion

Two new species of *Cytospora* are proposed from *Malusdomestica* and *M.sieversii* in Xinjiang, China, namely *C.hippophaopsis* and *C.shawanensis* based on morphological and phylogenetic studies. *C.hippophaopsis* is reported from cankers of *Malussieversii*, and *C.shawanensis* is discovered on the host *M.domestica*. Further pathogenicity tests confirmed their virulence to the branches of 13 varieties of *M.domestica* and *M.sieversii*. Hence, *Cytosporahippophaopsis* and *C.shawanensis* are two new apple canker pathogens well worth controlling.

*Cytosporahippophaopsis* from *Malussieversii* is generally more aggressive than *C.shawanensis* from *M.domestica* especially on Manpanzi (Fig. 5). In addition, on the varieties Erzizi, Hanfu, Manpanz, New Century, Oil Fruit, Sitagan and Red Star, *C.hippophaopsis* is also significantly more aggressive than *C.shawanensis*. While on the natural host, *M.sieversii*, *C.shawanensis* appears a bit more aggressive (not significant) than *C.hippophaopsis*. It is inferred that Manpanzi might be the original host of *C.hippophaopsis*.

In recent years, *M.sieversii* is facing an unprecedented crisis of survival, mainly in terms of its shrinking area, reduction of biological species, single community structure, and destruction of population renewal (Volk et al. 2013). According to previous studies in the wild, it was found that the problem of pests and diseases of *M.sieversii* is very prominent (Li and Zhang 2018). This investigation found that canker disease has occurred severely on *M.sieversii* in Ili and Tacheng areas. Previous studies on the pathogenic fungal species of *M.sieversii* have lacked the records of morphological characteristics and molecular data, but this study fills this gap. To further provide more effective information for the identification of canker disease pathogens, a large number of plant specimens should be collected from various hosts and from different regions in the future. The morphological and phylogenetic analyses reported in this study provide a theoretical basis for identifying *Cytospora* and for diagnosing canker diseases and lay the foundation for further pathogenicity studies and the protection of *M.sieversii* resources.

## Supplementary Material

XML Treatment for Cytospora hippophaopsis

XML Treatment for Cytospora shawanensis

## ﻿Additional information

### Author ORCIDs

Rong Ma https://orcid.org/0000-0002-9865-3047

### Data availability

All of the data that support the findings of this study are available in the main text.
